# Relative Age Effect in Physical Fitness of South Portugal Students between 10 and 18 Years Old

**DOI:** 10.3390/ijerph18116092

**Published:** 2021-06-05

**Authors:** Hugo Folgado, Jorge Bravo, Ana Quintas, Armando Raimundo, Bruno Gonçalves

**Affiliations:** 1 Departamento de Desporto e Saúde, Escola de Saúde e Desenvolvimento Humano, Universidade de Évora, Largo dos Colegiais, 7000-727 Évora, Portugal; jorgebravo@uevora.pt (J.B.); anaquintas_12@hotmail.com (A.Q.); ammr@uevora.pt (A.R.); bgoncalves@uevora.pt (B.G.); 2 Comprehensive Health Research Centre (CHRC), Universidade de Évora, Largo dos Colegiais, 7000-727 Évora, Portugal; 3 Portugal Football School, Portuguese Football Federation, 1495-433 Oeiras, Portugal

**Keywords:** age groups, physical education, RAE, muscular strength, aerobic fitness

## Abstract

Relative age is a phenomenon broadly studied in sport sciences. Youth sports participants born earlier in the selection year tend to present a maturational advantage over their peers. As it is also dependent on physical performance, older physical education students may also benefit from this effect in this school subject. The main goal of this manuscript was to determine whether the relative age effect is present within physical fitness outcomes of Portuguese children and adolescents. The physical–aerobic fitness, strength, flexibility and body composition of 885 students (490 females and 395 males) were collected and compared by quarters of birth, segmented by gender and age groups (10–12; 12–14; 14–16 and 16–18 years). The results reveal a moderate to small effect in physical fitness outcomes, with a trend for children and adolescents born in the early part of the year to present higher performance levels. These differences were more evident in ages closer to the physical maturational onset (12–14 y) and more apparent in male students. This physical fitness advantage may lead to a biased assessment and development of students born earlier in the year.

## 1. Introduction

Grouping children and adolescents into homogeneous chronological age cohorts is a common practice within educational and sport contexts. This procedure intends to balance learning opportunities and competitive experiences according to developmental differences for members of a particular age-grouped cohort [1]. However, an interval of up to one year of age may exist between children and adolescents competing or attending the same school year in the same age group [2]. Age grouping may confer an advantage to those born in the first months of the selection year due to individual differences in physical maturity and development, leading to unbalanced participation and attainment [2,3]. These potential cognitive and biological differences between children and adolescents within an age group are commonly reported as the relative age effect (RAE) [4,5,6,7,8].

Findings on the presence of RAEs in education reported that students born in the first months of an academic year exhibited higher school performance than those born later [9,10]. Two decades later, RAEs were identified in sport for the first time in an ice hockey cohort [11]. In agreement with the findings reported in education, the representativeness of ice hockey players born earlier in the same age group was higher than that in those born in the later months. Asymmetries in birth date and representation in sport are frequently reported. A meta-analysis reporting the relative age distribution of more than 130,000 sport participants showed an over-representation of youth athletes born in the first three months of a particular age-grouped cohort (first quartile) compared to those born in the last quartile [12]. Early maturation and consequent physical maturity of early-born athletes are commonly used to explain the RAE phenomenon [5]. The linking between early maturation and physical and body composition development may explain why older athletes within the same age group are more likely to succeed in youth sport.

Reports on RAEs in the education process have been widely reported, pointing out that later-born children and adolescents showed lower overall scores across subject areas. Furthermore, they were more often identified as having special needs and less likely to represent their school in sports competitions [13,14]. Prior research has also shown that RAEs are more prominent in early grades, especially cognitive performance [5]. However, when considering specific subjects where physical and body composition development plays a central role, the relationship between RAEs and grades is not linear. Several studies reported RAEs in physical education (PE), pointing to physical maturity as an advantage for those students born during the initial months of an academic year that is often mistaken with higher ability. Most studies are consensual about the effect of birth date, year group and sex on PE attainment, both in sports performance [1,15,16] and in written performance [16]. Similar findings were shown among lower and upper secondary school Norwegian adolescents, aged 15–16 and 18–19 years, respectively [17]. In their study, Aune et al. (2016) examined whether RAEs could be identified within non-competitive PE attainments in both sexes and if there was a change in RAE magnitude with age. The RAE was found in PE attainments for both sexes since 73% of the students who attained the highest marks were born in the first six months of the age group. Moreover, in lower secondary school, RAEs were larger in girls compared with boys [17]. Usual explanations to higher RAE prevalence in lower adolescence fall on the uneven onset of puberty, particularly within the PE subject, where the advantage of those who reach puberty earlier over their later-developing peers is most probable [18]. Although consensually accepted for boys [19], this explanation may not be evident for girls as their body changes during puberty could be detrimental to physical performance [20].

The Portuguese PE national curriculum includes, in addition to the essential sport modalities, an area for the assessment of physical fitness (PF) [21], aiming to encourage children and adolescents to improve their physical capacities, which in turn may promote skills acquisition, learning and positive attitudes [22]. Despite not counting for PE attainment, the PF assessment classifies students’ performances as being in the healthy fitness zone, needs improvement zone or needs improvement-health risk zone [23], having a potential impact on students’ self-esteem [24], psychological and general health [25,26]. PF outcomes tend to be biased and affected by children’s and adolescents’ relative age [1,15,16]; therefore, it is expected to find RAEs in Portuguese students from the mistake between early physical maturity and higher PF [2,17].

The overall purpose of the current study was to determine whether the RAE is present within PF outcomes in Portuguese children and adolescents. Furthermore, in the case of the RAE presence in Portuguese children and adolescents, we intended to examine whether these are common to boys and girls, independently of the age group, and whether the effects are cross-sectional to all age groups or exclusive to the youngest ones. It was hypothesized that youths born earlier in the selection year might present a maturational advantage over their peers as in sport settings.

## 2. Materials and Methods

### 2.1. Design and Participants

This study followed an observational cross-sectional design and is part of a nationwide survey examining a representative sample of the Portuguese population. Participants were 885 children and adolescents (490 girls, 55.4%) aged 10 to 18 (14.5 ± 2.5) recruited from Portuguese mainland public schools through proportional stratified random sampling, considering the administrative regions of Alentejo and Algarve (South of Portugal) exclusively. The population was selected through proportionate stratified random sampling, considering the location (region) and the number of students by age and sex. Schools were randomly selected within each region and agreed to participate in the study. Finally, the sample was balanced by a weight factor to adjust for the distribution of the Portuguese population in the schools and to guarantee each age group’s representativeness (age and sex). The minimum representative sample size was 881 children and adolescents, calculated through epidemiologic statistical software. Inclusion criteria considered children aged between 9.50 and 18.49 without impediments to participate in the physical education classes.

Data collection took place between March 2017 and November 2018 during morning physical education classes by investigators trained for the collection procedures. School-based testing was used, with a mobile test team that performed testing under a physical education teacher’s supervision. Testing was conducted throughout the entire school year to prevent seasonal bias on the assessed parameters. The daily order of testing was standardized to minimize between-test interaction. The study was conducted in full compliance with the Helsinki Declaration and approved by the Faculty of Human Kinetics Ethics Committee, University of Lisbon (ID number: 25/2020). Written informed consent was obtained from all parents or legal guardians, and a code was assigned to each participant during data collection to maintain anonymity.

### 2.2. Body Composition

Weight and height were measured according to standardized procedures [27]. Height was measured to the nearest 0.1 cm with a stadiometer (model 220 SECA, Hamburg, Germany) and weight to the nearest 0.01 kg on an electronic scale (model 799 SECA, Hamburg, Germany) while participants were wearing minimal clothing and without shoes. Body mass index (BMI) was calculated as body mass/height^2^ (kg/m^2^). Waist circumference was measured following standardized procedures described elsewhere [28], with a flexible anthropometric tape (SECA, Hamburg, Germany) positioned parallel to the floor and immediately above the iliac crest at the end of minimal respiration, with the participant in a standing position. The waist circumference value was registered to the nearest 0.1 cm.

### 2.3. Physical Fitness

A subset of tests from the FITNESSGRAM test battery was used to assess PF, namely, aerobic fitness, strength and endurance and flexibility. A complete description of all the tests used to access PF and how scores are assigned can be found elsewhere [23]. Aerobic fitness was assessed by the Progressive Aerobic Cardiovascular Endurance Run (PACER) test and scored based on the number of completed courses (laps). Arms and abdominal strength and endurance were evaluated by the number of completed repetitions on the push-up and curl-up tests (reps), respectively. The best value of each leg was recorded from the sit and reach test to assess trunk flexibility (cm).

### 2.4. Data Analysis

To examine the birth date distribution, participants were grouped into four quarters of three months each (equivalent to the quarters of the year) according to their date of birth. The first quarter (Q1) comprised January, February and March, the second quarter (Q2) included April, May and June, the third quarter (Q3) included July, August and September and the fourth quarter (Q4) comprised October, November and December. The cohort spanned eight years, the participants being allocated to Group 1 (10 to 12 y), Group 2 (12 to 14 y), Group 3 (14 to 16 y) or Group 4 (16 to 18 y) for females and males.

Statistical analysis was conducted using The Jamovi Project [29]. Descriptive analysis is presented on Table 1 and Table 2, with mean and standard deviation (Mean ± SD). A one-way analysis of variance (ANOVA) was performed to identify differences in the considered variables across birthday quarters for females and males. For each ANOVA, partial eta-squared (*η*_p_^2^) was calculated, and values of 0.01, 0.06 and above 0.14 were considered small, medium and large, respectively [30]. Statistical significance was set at *p* < 0.05. Complementarily, to overcome the shortcomings associated with traditional N–P null hypothesis significance testing, the standardized Cohen *d*, with a 95% confidence interval as the effect size (ES), was applied to identify pairwise differences [31,32,33]. Thresholds for effect size statistics were as follows: 0.0–0.19, trivial; 0.20–0.59, small; 0.6–1.19, moderate; 1.2–1.9, large; and ≥2.0, very large [33]. 

## 3. Results

**Figure 1:**
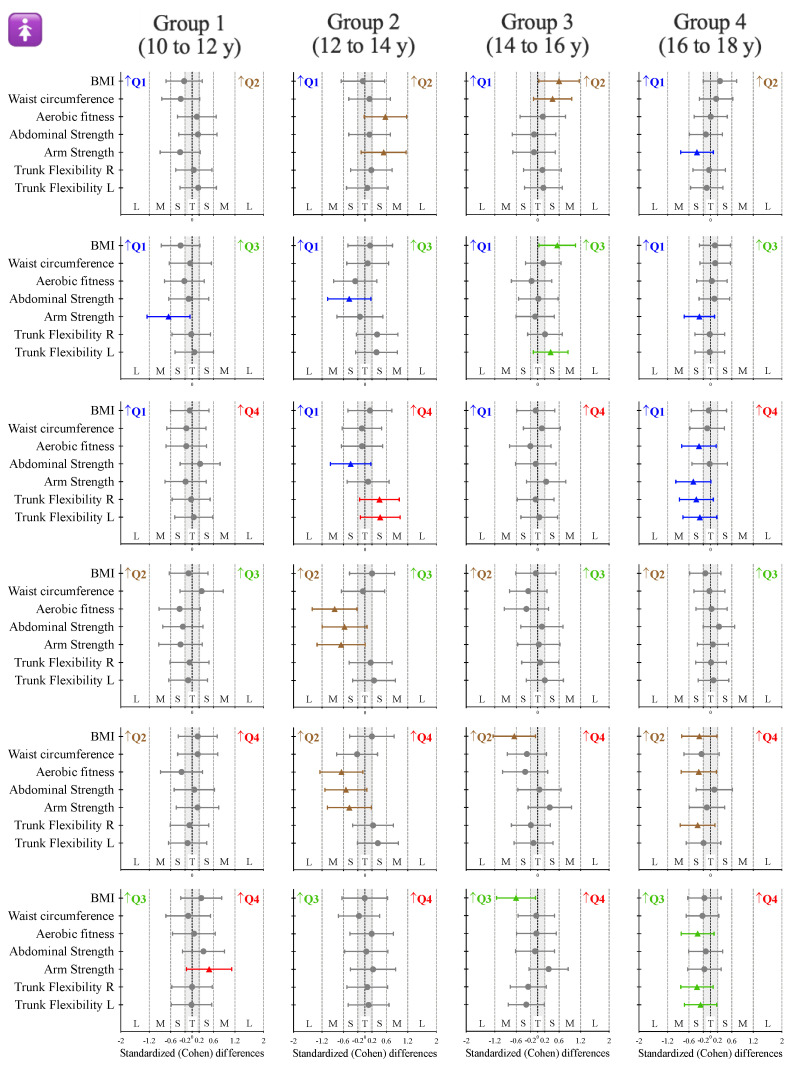
Standardized (Cohen) differences in the considered variables according to the age group for female participants. Error bars indicate uncertainty in the true mean changes with 95% confidence intervals. Abbreviations: Q1 = first quarter, participants born in January, February and March; Q2 = second quarter, participants born in April, May and June; Q3 = third quarter, participants born in July, August and September; Q4 = fourth quarter, participants born in October, November and December. ↑ = means that the participants from that quarter presented higher values. Gray error bars denote unclear results.

The inferential analysis between quarters and according to the age group for female participants is presented in Table 1. Complementarily, Figure 1 shows the standardized (Cohen) differences for the pairwise comparisons. Non-significant differences were identified for age group 1 (10 to 12 y). Age group 2 (12 to 14 y) showed differences for the aerobic fitness test (F = 2.9, *p* = 0.042, *η*_p_^2^ = 0.058), where Q2 presented higher values compared to Q3 and Q4 (both with a moderate ES). Despite the non-significant results, the same ESs were identified in abdominal and arm strength. In group 3 (14 to 16 y), the BMI showed significant differences (F = 3.2, *p* = 0.028, *η*_p_^2^ = 0.084), where the participants born in Q2 and Q3 showed small to moderate higher values compared to Q1 and Q4. For group 4 (16 to 18 y), while no statistical differences were reported, Q1 participants presented small higher values in aerobic fitness (compared to Q4), arm strength (compared to Q2, Q3 and Q4) and both right and left trunk flexibility (compared to Q4) (see Figure 1).

**Figure 2:**
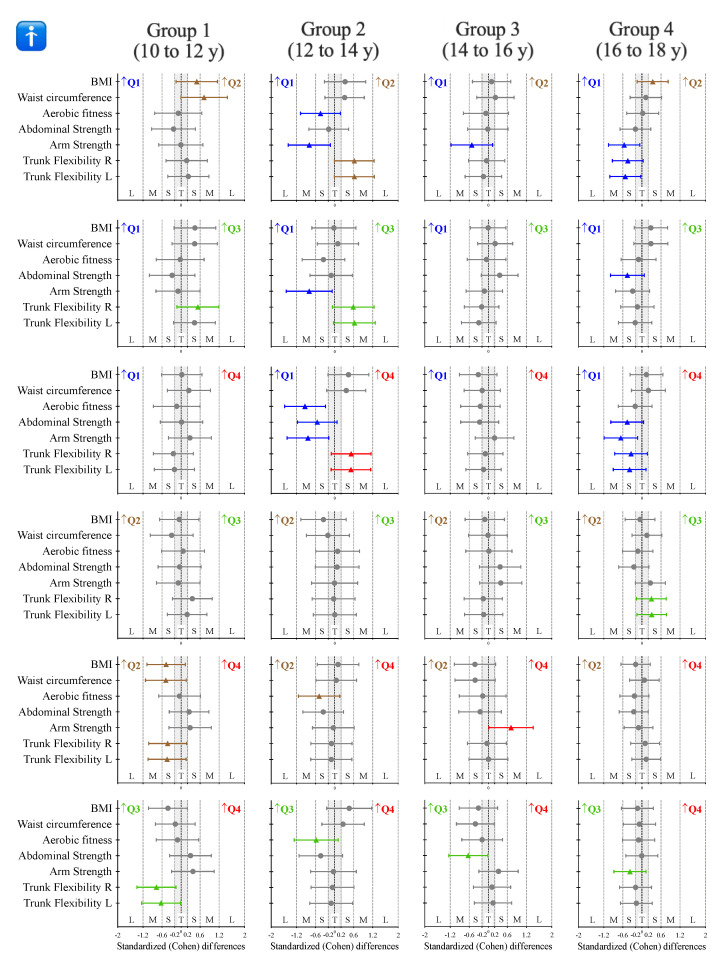
Standardized (Cohen) differences in the considered variables according to the age group for male participants. Error bars indicate uncertainty in the true mean changes with 95% confidence intervals. Abbreviations: Q1 = first quarter, participants born in January, February and March; Q2 = second quarter, participants born in April, May and June; Q3 = third quarter, participants born in July, August and September; Q4 = fourth quarter, participants born in October, November and December. ↑ = means that the participants from that quarter presented higher values. Gray error bars denote unclear results.

The inferential analysis between quarters and according to the age group for male participants is presented in Table 2. Complementarily, Figure 2 shows the standardized (Cohen) differences for the pairwise comparisons. Non-significant differences were identified for age group 1 (10 to 12 y). Age group 2 (12 to 14 y) showed differences for aerobic fitness (F = 3.0, *p* = 0.036, *η*_p_^2^ = 0.112), where Q1 presented higher values compared to Q4 (small ES), and for arm strength (F = 3.18, *p* = 0.030, *η*_p_^2^ = 0.126), where Q1 presented higher values compared to Q2, Q3 and Q4 (moderate ES). Non-significant differences were identified for age group 3 (14 to 16 y). Finally, while non-significant differences were also reported for group 4 (16 to 18 y), a clear trend was identified. Participants born in Q1 presented small to moderate higher values in abdominal strength, arm strength and both right and left trunk flexibility (see Figure 2).

## 4. Discussion

The main goal of the current study was to determine whether the RAE is present within PF outcomes in Portuguese children and adolescents. The results show that, despite having a moderate to small effect, there are differences in PF outcomes, with a trend for children and adolescents born in the early part of the year to present higher levels of performance. These results are in line with existing research that focus on RAE effects on performance in several contexts of practice, namely, youth competitive sport, but also in physical education at schools. In different competitive team sports, players born in the first quarters of the year tend to be over-represented in their teams, and to present higher levels of physical performance in different age groups [34,35,36]. These higher levels of PF are identified as one of the main mechanisms to explain the RAE in sport, as more mature athletes, born in the earlier parts of the year, tend to exhibit advanced physical characteristics compared to their younger peers [37]. As maturational differences reduce with age progression, the maintenance of representation and performance differences are justified by a positive feedback process, as selected athletes benefit from better training and more opportunities to compete, leading to higher levels of development [37]. In educational settings, despite the inexistence of “selection” procedures, with students from the same age group typically following the same curriculum, there is also evidence of the RAE amongst students, particularly in PE. In fact, students born in the early part of the year tend to be more represented in sports participation [1] and to present higher marks in PE [17]. However, studies addressing the RAE in physical education tend to lack objective functional fitness testing results, leaving some room for speculation when analyzing their results, as they may be related to physical maturation, but also to psychological or pedagogical factors [38]. This study highlights the differences in PF results of children and adolescents born in earlier or later parts of the year, helping to justify the RAE in those scenarios.

Considering specific age groups, few differences between quarters were identified in the younger 10 to 12 years old group. When identified, differences were mostly present in the male participants and for BMI, waist circumference and flexibility, and without presenting a clear trend between participants born in the earlier and later parts of the year. The younger ages of this group, despite augmenting the relative age difference within the participants, also place them further away from their maturational offset. This difference reduces possible maturational advantages for participants born at earlier moments of the year, diminishing their physical differences. Comparable results were identified in competitive sports, where younger age football players (10–12 y) born in the earlier part of the year, despite being over-represented in their teams, presented fewer PF differences to players born in the later part of the year than older age groups [39,40]. In the present study, this effect could be augmented given that no previous selection was performed on the participants.

The 12 to 14 years old group evidenced the most relevant effect of relative age in the PF results of the participants. Generally, students born in the earlier part of the year, either female or male, presented higher results of aerobic fitness, abdominal strength and arm strength. As stated earlier, this group includes a majority of students that are closer to their puberty onset and peak height velocity [41]. This factor potentially amplifies the RAE, as students born in the earlier part of the year benefit from a maturational advantage in their PF results. In competitive team sports, football players with an early maturational status tend to be more often selected for football academies when reaching the lower interval of this age group (12 years), suggesting that maturational differences are more relevant as players increase in their age [42]. A study from Hill et al. [42] highlighted that despite the presence of the RAE for all age groups of a football academy, a maturational bias in the player selection was only present from early adolescence. These results support the idea that students born in the early part of the selection year tend to benefit from a chronological advantage at their earlier years, where the relative difference is more noticeable, and that they benefit from an earlier maturational onset when they are closer to their peak height velocity. Additionally, in fact, preschool children between 3 and 5 years old do tend to present marked differences in their PF results per quarter, with children born in the first quarters of the year exhibiting higher levels of performance [43]. However, in a study by Cupeiro et al. [43], the researchers also identified an attenuation in the magnitude of the differences between quarters for older groups in some of the tests, suggesting that their physical advantage decreases as the relative age difference also reduces. 

Finally, the last two age groups present different results. The 14 to 16 years old age group presents few relevant differences, and without a clear trend between quarters, for both genders. As for the 16 to 18 years old age group, there are no statistically significant results, but there seems to be a relevant effect in strength and flexibility for both groups and in aerobic fitness in female students. In all cases, participants born in the first quarters of the year presented better PF results than their peers born later in the year. These were somewhat unexpected results, particularly for the female students. Typically, after reaching their maturity, female students tend to dimmish their levels of physical activity and even reduce their level of PF [44]. In our study, given the cross-sectional analysis, it is possible that the students evaluated within this age group, and particularly those born in the first quarters of the year, were more physically active and further enrolled in competitive sports than their younger peers. Still, our results do adhere to previous research of the RAE effect in female sports, where this effect is more present in younger age groups, with a lower magnitude difference for post-adolescent age groups [45]. 

Considering the separate analysis of female and male students, despite a similar trend between age groups, with students born in the first quarters of the year showing better PF results in most of the groups, results show that male students typically presented a higher magnitude of the standardized differences than female students. Given the more intense growth of males during the onset of their maturational biomarkers, such as peak height velocity [46], their physical advantage seems to be more pronounced at the ages near this onset, when compared to students born in the later part of the year. Some research conducted in PE environments seems to support this idea, revealing a more intense RAE for male than for female students. Results suggest that male students born in the first quarters of the year tend to exhibit higher differences in PE marks and in PF results than their peers born in the later part of the year [2,17,47].

The presented results highlight the existence of the RAE in PE for both male and female students. Despite the small to moderate differences between quarters, older students may benefit from their development advantage, leading to an unfair process in their development and assessment. Given the everyday use for physical evaluation in PE, where both fitness and body composition are measured, existing protocols should also account for introducing the decimal age and quarter of birth as a weighing factor. The simple information of these aspects seems to have a beneficial effect in the selection bias in competitive sports [48] and could balance student development in the educational environment.

Overall, the results from the present study add important insights regarding the maturational advantage promoted by the RAE. Nevertheless, this study reports on south Portugal students, and thus attention should be taken to generalize the results to other regions and countries. Additionally, educational organization is country-specific, and this issue should be considered in future studies. Furthermore, future researchers should aim to control relevant aspects that may influence the results, namely, identifying students who participate in competitive sport outside the school environment. As sports participation contributes significantly to participants’ physical development, students enrolled in individual or collective sports may present higher physical fitness results.

## 5. Conclusions

This study revealed that Portuguese children’s and adolescent’s PF results are affected by the RAE, as students born in the first quarters of the year present a normally moderate advantage over their later-born peers. These differences are evident in ages closer to the physical maturational onset and more apparent in male students. Teachers and educational staff should be aware of the effect of the RAE in physical education, as the PF advantage may lead to a biased assessment and development of students born earlier in the year. 

## Figures and Tables

**Table 1:** Characterization and inferential analysis of the considered variables according to the age group for female participants.

Variables		Q1		Q2		Q3		Q4	
		N	Mean ± SD	N	Mean ± SD	N	Mean ± SD	N	Mean ± SD
Group 1 (10 to 12 y)	Body mass index	31	19.6 ± 4.4	30	18.7 ± 4.0	24	18.4 ± 3.1	24	19.4 ± 3.6
	Waist circumference	30	67.2 ± 10.1	27	64.3 ± 10.8	18	66.8 ± 8.4	23	65.8 ± 5.7
	Aerobic fitness	29	28.9 ± 15.4	25	30.7 ± 13.4	23	26.0 ± 12.7	21	26.7 ± 10.8
	Abdominal strength	28	33.8 ± 23.3	27	37.9 ± 25.7	23	31.4 ± 25.9	23	39.5 ± 26.6
	Arm strength	27	8.4 ± 5.9	24	6.2 ± 7.9	19	4.1 ± 5.0	21	7.2 ± 6.5
	Trunk flexibility (right)	31	23.0 ± 7.5	30	23.4 ± 6.9	24	22.8 ± 9.2	25	22.8 ± 9.1
	Trunk flexibility (left)	31	21.9 ± 7.5	30	23.3 ± 6.6	24	22.4 ± 9.1	25	22.2 ± 10.3
Group 2 (12 to 14 y)	Body mass index	22	19.5 ± 3.2	21	19.3 ± 3.4	19	20.1 ± 4.5	20	20.0 ± 4.5
	Waist circumference	25	67.1 ± 9.4	22	68.4 ± 12.1	21	67.9 ± 10.7	27	66.2 ± 7.2
	Aerobic fitness	24	28.1 ± 10.5	22	34.3 ± 10.5 ^d,e^	20	25.2 ± 8.1	23	27.2 ± 13.3
	Abdominal strength	25	36.0 ± 24.5	22	38.9 ± 24.9	19	26.3 ± 18.7	25	27.2 ± 19.2
	Arm strength	22	7.6 ± 8.8	19	12.3 ± 10.9	17	6.4 ± 5.8	24	8.5 ± 9.0
	Trunk flexibility (right)	25	18.5 ± 15.0	22	20.6 ± 11.8	22	22.5 ± 10.5	27	23.4 ± 9.6
	Trunk flexibility (left)	25	18.8 ± 11.9	22	19.5 ± 11.3	22	22.3 ± 10.8	27	23.4 ± 8.8
Group 3 (14 to 16 y)	Body mass index	31	20.6 ± 4.2 ^a,b^	21	22.9 ± 3.8 ^e^	30	22.7 ± 3.92 ^f^	25	20.4 ± 2.9
	Waist circumference	31	68.0 ± 9.0	25	72.6 ± 14.4	33	69.8 ± 12.8	28	69.3 ± 6.3
	Aerobic fitness	23	33.7 ± 15.4	17	35.5 ± 14.4	27	31.4 ± 10.7	24	31.0 ± 11.6
	Abdominal strength	24	36.8 ± 22.6	19	34.7 ± 18.8	28	37.2 ± 21.6	25	35.7 ± 20.2
	Arm strength	27	7.9 ± 7.1	19	7.3 ± 4.5	28	7.5 ± 5.5	25	9.4 ± 6.8
	Trunk flexibility (right)	32	22.8 ± 9.3	25	24.1 ± 7.8	35	24.7 ± 8.0	28	22.3 ± 11.6
	Trunk flexibility (left)	32	20.9 ± 9.8	25	22.5 ± 8.3	35	24.5 ± 8.4	28	21.4 ± 12.2
Group 4 (16 to 18 y)	Body mass index	36	21.3 ± 4.7	36	22.5 ± 4.2	48	21.9 ± 3.3	29	21.1 ± 5.5
	Waist circumference	37	67.8 ± 9.5	35	69.4 ± 11.4	51	69.1 ± 8.5	30	66.9 ± 11.2
	Aerobic fitness	38	33.9 ± 11.2	35	34.0 ± 11.0	49	34.4 ± 15.3	30	29.9 ± 10.7
	Abdominal strength	38	40.1 ± 22.6	35	37.2 ± 18.7	49	42.4 ± 22.4	27	39.6 ± 23.4
	Arm strength	40	14.7 ± 25.6	36	9.3 ± 6.0	48	10.3 ± 7.4	28	7.9 ± 6.6
	Trunk flexibility (right)	40	26.4 ± 11.3	36	26.0 ± 10.2	51	26.2 ± 11.0	31	21.9 ± 13.4
	Trunk flexibility (left)	40	26.0 ± 11.2	36	24.8 ± 10.9	51	25.8 ± 11.5	31	22.5 ± 14.2

Pairwise differences (*p* < 0.05) are reported as: ^a^ Q1 vs. Q2; ^b^ Q1 vs. Q3; ^c^ Q1 vs. Q4; ^d^ Q2 vs. Q3; ^e^ Q2 vs. Q4; ^f^ Q3 vs. Q4.

**Table 2:** Characterization and inferential analysis of the considered variables according to the age group for male participants.

Variables		Q1		Q2		Q3		Q4	
		N	Mean ± SD	N	Mean ± SD	N	Mean ± SD	N	Mean ± SD
Group 1 (10 to 12 y)	Body mass index	17	17.8 ± 2.4	21	19.3 ± 4.0	20	19.1 ± 2.9	23	17.9 ± 2.4
	Waist circumference	14	62.5 ± 6.4	17	68.6 ± 11.0	18	66.1 ± 9.3	23	64.6 ± 5.8
	Aerobic fitness	12	36.3 ± 10.5	18	34.7 ± 15.1	17	35.8 ± 18.0	19	33.9 ± 21.0
	Abdominal strength	15	38.3 ± 20.6	19	33.3 ± 15.6	16	32.2 ± 20.2	22	38.7 ± 26.4
	Arm strength	16	8.8 ± 7.5	17	8.7 ± 5.9	17	8.0 ± 8.6	19	10.9 ± 9.0
	Trunk flexibility (right)	17	16.2 ± 9.0	21	17.5 ± 7.3	20	20.2 ± 6.1	24	14.4 ± 7.3
	Trunk flexibility (left)	17	16.1 ± 8.6	21	17.8 ± 7.0	20	19.2 ± 6.0	24	14.6 ± 7.7
Group 2 (12 to 14 y)	Body mass index	20	19.2 ± 3.9	18	20.7 ± 4.8	14	19.1 ± 5.0	19	21.2 ± 4.0
	Waist circumference	23	69.5 ± 8.2	19	73.3 ± 14.0	16	70.7 ± 13.3	20	73.9 ± 12.8
	Aerobic fitness	22	50.5 ± 18.2 ^c^	19	41.7 ± 22.5	15	43.5 ± 15.4	19	32.1 ± 21.1
	Abdominal strength	22	41.7 ± 23.9	19	37.4 ± 22.7	15	39.2 ± 27.6	20	29.2 ± 17.1
	Arm strength	20	15.9 ± 11.8 ^a,b,c^	18	8.5 ± 7.5	13	8.5 ± 6.3	19	8.1 ± 9.4
	Trunk flexibility (right)	23	11.9 ± 12.8	19	17.7 ± 7.8	16	17.4 ± 8.7	19	16.8 ± 6.1
	Trunk flexibility (left)	23	10.6 ± 14.1	19	16.9 ± 8.5	16	16.9 ± 8.1	19	15.8 ± 6.2
Group 3 (14 to 16 y)	Body mass index	27	21.2 ± 4.2	18	21.6 ± 4.3	24	21.1 ± 4.0	20	19.9 ± 2.8
	Waist circumference	29	69.8 ± 16.6	19	72.9 ± 14.0	24	72.8 ± 12.3	21	67.1 ± 11.9
	Aerobic fitness	23	59.5 ± 25.0	12	57.8 ± 25.6	20	58.2 ± 19.5	19	53.7 ± 23.1
	Abdominal strength	25	48.2 ± 22.0	16	47.9 ± 23.3	23	56.2 ± 21.4	20	42.3 ± 22.0
	Arm strength	25	16.5 ± 9.6	15	11.3 ± 8.1	23	15.3 ± 9.3	19	18.5 ± 12.9
	Trunk flexibility (right)	30	19.3 ± 9.7	20	18.8 ± 6.2	24	17.3 ± 12.5	22	18.4 ± 7.6
	Trunk flexibility (left)	30	18.6 ± 10.4	20	17.0 ± 7.3	24	15.5 ± 12.9	22	17.1 ± 9.1
Group 4 (16 to 18 y)	Body mass index	27	21.6 ± 1.7	44	22.9 ± 4.1	30	22.7 ± 4.5	32	22.1 ± 3.9
	Waist circumference	25	72.0 ± 15.9	44	73.7 ± 13.2	30	75.8 ± 12.5	31	74.8 ± 12.0
	Aerobic fitness	24	66.0 ± 22.0	44	66.5 ± 21.9	28	63.6 ± 26.5	33	61.2 ± 19.7
	Abdominal strength	27	58.6 ± 20.8	44	53.8 ± 21.1	29	48.0 ± 27.4	33	47.9 ± 23.4
	Arm strength	26	25.4 ± 13.6 ^a^	45	19.3 ± 8.8	29	22.2 ± 12.2	33	18.2 ± 9.5
	Trunk flexibility (right)	27	24.4 ± 10.3	45	18.4 ± 15.3	30	22.5 ± 14.1	33	19.8 ± 12.7
	Trunk flexibility (left)	27	24.0 ± 10.6	45	17.1 ± 14.5	30	21.2 ± 14.2	33	18.9 ± 12.8

Pairwise differences (*p* < 0.05) are reported as: ^a^ Q1 vs. Q2; ^b^ Q1 vs. Q3; ^c^ Q1 vs. Q4.

## Data Availability

The data that support the findings of this study are available from the corresponding author, H.F., upon reasonable request.
